# Dr. Battey

**Published:** 1895-11

**Authors:** 


### DR. BATTEY.

We are pleased to say that the press dispatches have been premature in announcing the death of Dr. Battey. As we go to press we learn that, while his illness has been quite serious and his health much impaired, there is at present some improvement in his condition. We sincerely trust that this will continue.

Medical journals which have been misled into publishing the death of Dr. Battey will please make proper correction.

